# Genetic Determinants of Intrinsic Antibiotic Tolerance in Mycobacterium avium

**DOI:** 10.1128/Spectrum.00246-21

**Published:** 2021-09-15

**Authors:** William M. Matern, Harley Parker, Carina Danchik, Leah Hoover, Joel S. Bader, Petros C. Karakousis

**Affiliations:** a High-Throughput Biology Center, Department of Biomedical Engineering, Johns Hopkins University School of Medicine, Baltimore, Maryland, USA; b Center for Systems Approaches to Infectious Diseases (C-SAID), Johns Hopkins University School of Medicine, Baltimore, Maryland, USA; c Center for Tuberculosis Research, Department of Medicine, Johns Hopkins University School of Medicine, Baltimore, Maryland, USA; d Department of International Health, Johns Hopkins Bloomberg School of Public Health, Baltimore, Maryland, USA; Quest Dianostics Nicols Institute

**Keywords:** DNA sequencing, bioinformatics, mechanisms of action, molecular genetics, persistence, tolerance

## Abstract

The Mycobacterium avium complex (MAC) is one of the most prevalent causes of nontuberculous mycobacteria pulmonary infection in the United States, and yet it remains understudied. Current MAC treatment requires more than a year of intermittent to daily combination antibiotic therapy, depending on disease severity. In order to shorten and simplify curative regimens, it is important to identify the innate bacterial factors contributing to reduced antibiotic susceptibility, namely, antibiotic tolerance genes. In this study, we performed a genome-wide transposon screen to elucidate M. avium genes that play a role in the bacterium’s tolerance to first- and second-line antibiotics. We identified a total of 193 unique M. avium mutants with significantly altered susceptibility to at least one of the four clinically used antibiotics we tested, including two mutants (in DFS55_00905 and DFS55_12730) with panhypersusceptibility. The products of the antibiotic tolerance genes we have identified may represent novel targets for future drug development studies aimed at shortening the duration of therapy for MAC infections.

**IMPORTANCE** The prolonged treatment required to eradicate Mycobacterium avium complex (MAC) infection is likely due to the presence of subpopulations of antibiotic-tolerant bacteria with reduced susceptibility to currently available drugs. However, little is known about the genes and pathways responsible for antibiotic tolerance in MAC. In this study, we performed a forward genetic screen to identify M. avium antibiotic tolerance genes, whose products may represent attractive targets for the development of novel adjunctive drugs capable of shortening the curative treatment for MAC infections.

## INTRODUCTION

Nontuberculous mycobacteria (NTM) are found ubiquitously in the environment, and several species can cause disease especially in the elderly, those with preexisting lung disease, and the immunocompromised, including those infected with HIV (1–4). The Mycobacterium avium complex (MAC), a group of 12 related, slow-growing mycobacteria with Mycobacterium intracellulare and Mycobacterium avium as the most prevalent species, accounts for the majority of pulmonary infections due to NTM in the United States (5, 6). Although the true incidence of pulmonary MAC infections in the United States is not known, a study in Oregon reported 4.8 cases per 100,000 person-years in 2012 (7). Winthrop et al. estimated the annual incidence of NTM infections in the United States to be 4.73 cases per 100,000 person-years (8), and the NTM Network European Trials Group has reported that MAC accounts for 52% of NTM isolates in the United States and Canada (9), suggesting that the annual incidence of MAC in the United States may be closer to ∼2.5 per 100,000 person-years.

The current treatment for MAC comprises a combination of multiple antibiotics given for at least 12 months following the conversion of sputum cultures from positive to negative. Since sputum culture conversion occurs in ∼50% of cases after 5 months of antibiotic therapy, a typical patient receives a minimum of 15 to 18 months of treatment (10, 11). Macrolide-susceptible MAC infection is usually treated with at least three antibiotics, including a macrolide (azithromycin or clarithromycin), a rifamycin (rifampin or rifabutin), and ethambutol, either intermittently (three times weekly) or daily for severe fibronodular or cavitary disease. If the infecting MAC strain is macrolide-resistant or the patient is unable to take the first-line regimen, alternative antibiotics, such as moxifloxacin, clofazimine, or linezolid, are often used (11, 12).

The lengthy and complicated treatment course required to eradicate MAC infection has been attributed to the presence of persistent organisms, which exhibit reduced susceptibility, or tolerance, to antibiotics (13). Unlike antibiotic resistance, which results from a heritable genetic alteration permitting continued bacterial growth in the presence of antibiotic concentrations exceeding the MIC, antibiotic tolerance is a transient, nonheritable phenotype without associated change in the MIC. The term antibiotic tolerance was originally coined in 1970 by Tomasz et al. to denote the ability of bacteria to withstand the bactericidal activity of antibiotics, especially of cell wall-active agents, primarily by reducing their replication rate (14). In the intervening decades, additional mechanisms have been proposed to mediate bacterial antibiotic tolerance, including biofilm formation (15, 16), induction of the stringent response (17–20) or efflux pumps (21–23), and altered metabolism (24, 25). Following ingestion by macrophages, MAC members acquire an antibiotic-tolerant phenotype within the arrested phagosome (26). Moreover, various stress conditions, including nutrient starvation, low pH, and hypoxia, induce a nonreplicative, antibiotic-tolerant state (24), which is characterized by transcriptional changes (24, 25), leading to altered cell wall membrane permeability (3) and an increased expression of efflux pumps (21). This stress-induced adaptation of MAC is accompanied by dramatically reduced metabolism, with a shift to the glyoxylate shunt, stabilization of the mycolate pool, and a switch to transcription of only essential genes (25). Additionally, the glyoxylate shunt enzyme isocitrate lyase is critical for the long-term survival of the related pathogen Mycobacterium tuberculosis in host tissues (27) and can be used in a reductive amination pathway to produce NAD, which may serve as an alternative energy source in a nonreplicative state and under anaerobic conditions (28, 29). However, the molecular mechanisms driving antibiotic tolerance in MAC remain poorly understood.

Transposon insertion sequencing (Tn-seq) is a powerful technique for determining bacterial genotype-phenotype relationships, particularly specific bacterial genes that are required for growth and/or survival under controlled stress conditions (30–33). Modifications of this technique have been used to define essential genes for *in vitro* growth of M. tuberculosis (34) and M. avium (35). Xu et al. screened a saturated transposon mutant library in the presence of partially inhibitory concentrations of various antibiotics with diverse mechanisms of action to identify genetic determinants of intrinsic antibiotic susceptibility in M. tuberculosis (36). In the current study, we used a similar approach to identify genes responsible for the intrinsic tolerance of M. avium to the antibiotics clarithromycin (CLR), rifabutin (RFB), moxifloxacin (MOX), and ethambutol (EMB). The hits we have identified may serve as targets for the development of novel antibiotics, with the objective of shortening the duration of curative treatment for MAC.

## RESULTS

### Effects of antibiotics on bacterial growth at the population level.

To monitor the effects of individual antibiotics on the entire bacterial population, we measured CFU and optical density at 600 nm (OD_600_) during antibiotic exposure. CFU values obtained following antibiotic exposure for 0, 12, and 48 h are provided in Fig. S2 in the supplemental material. OD_600_ values are provided in Fig. S3 in the supplemental material. The same no-drug (vehicle) control data appear in all four plots (performed in triplicate). Notably, the no-drug control curve has an inflection at the 12-h time point. Truly logarithmic growth should appear as a straight line on this plot. Light microscopy of unstained samples of the no-drug control cultures revealed clumps of approximately 5 bacteria (data not shown), likely accounting for the inflection point. Bacterial clumping was likely also present in the antibiotic-containing tubes, although these were not specifically examined. Additionally, we observed that the OD curve increased for most samples from 0 h to 12 h, whereas the CFU remained stable or declined over this time. In our view, this discrepancy between OD and CFU is most likely accounted for by growth of new bacteria in the context of larger bacterial clumps, which scatter more light (increased OD) but may not increase the CFU (although viable bacterial cells per clump will increase). The presence of clumping is unlikely to affect the results of the screen, as there is no reason to suspect that individual transposon mutants were disproportionately distributed among the clumps.

Applying the set of criteria for selecting samples for processing as described in the Methods, we prepared libraries and sequenced both time points at the following concentrations: 0.54 and 5.4 μg/ml CLR, 0.1 and 1.0 μg/ml MOX, 0.063 and 0.63 μg/ml RFB, and 0.21 and 2.1 μg/ml EMB. The colored arrows in Fig. S2 and S3 indicate the time points sequenced.

### Identification of mutants with altered antibiotic susceptibility.

**FIG 1:**
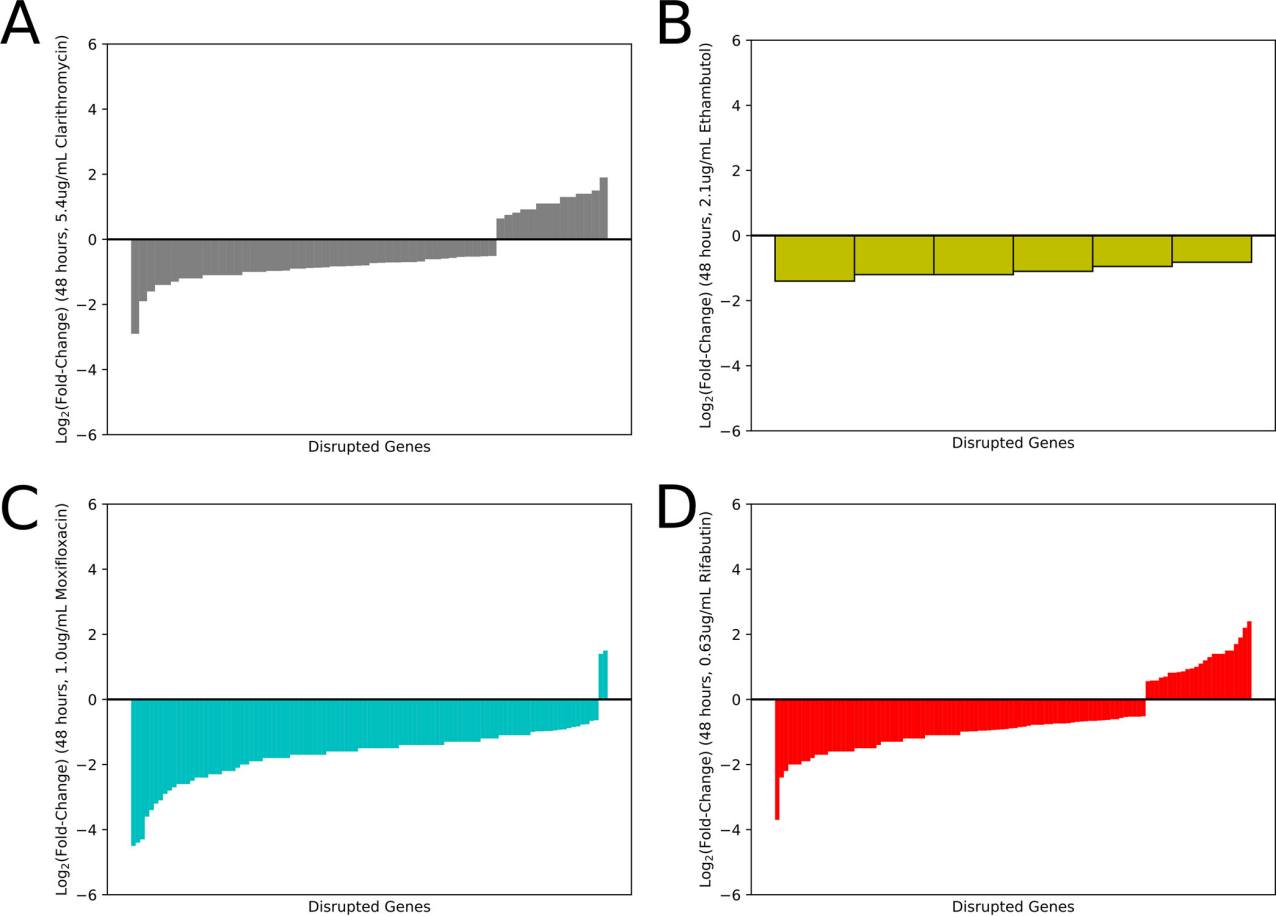
Bar chart showing the effect size of each statistically significant mutant. Each bar represents a single gene. A negative value represents a hypersusceptible mutant, while a positive value signifies that a mutant is less susceptible (hypertolerant) to the antibiotic. (A) Clarithromycin, (B) ethambutol, (C) moxifloxacin, (D) rifabutin.

A total of 161 mutants showed increased susceptibility and 32 mutants showed reduced susceptibility to at least 1 of the 4 antibiotics. A total of 46 mutants were hypersusceptible and 14 mutants were hypertolerant to CLR. Six mutants were found to be hypersusceptible to EMB, while no mutants were hypertolerant to this antibiotic. The MOX screen revealed 103 hypersusceptible and 2 hypertolerant mutants. A total of 84 mutants were found to be hypersusceptible and 108 mutants were hypertolerant to RFB. Effect sizes (after 48 hours of exposure) for mutants with significantly altered antibiotic susceptibility are plotted in Fig. 1 and summary data are provided in Tables S3 to S6 in the supplemental material.

**FIG 2:**
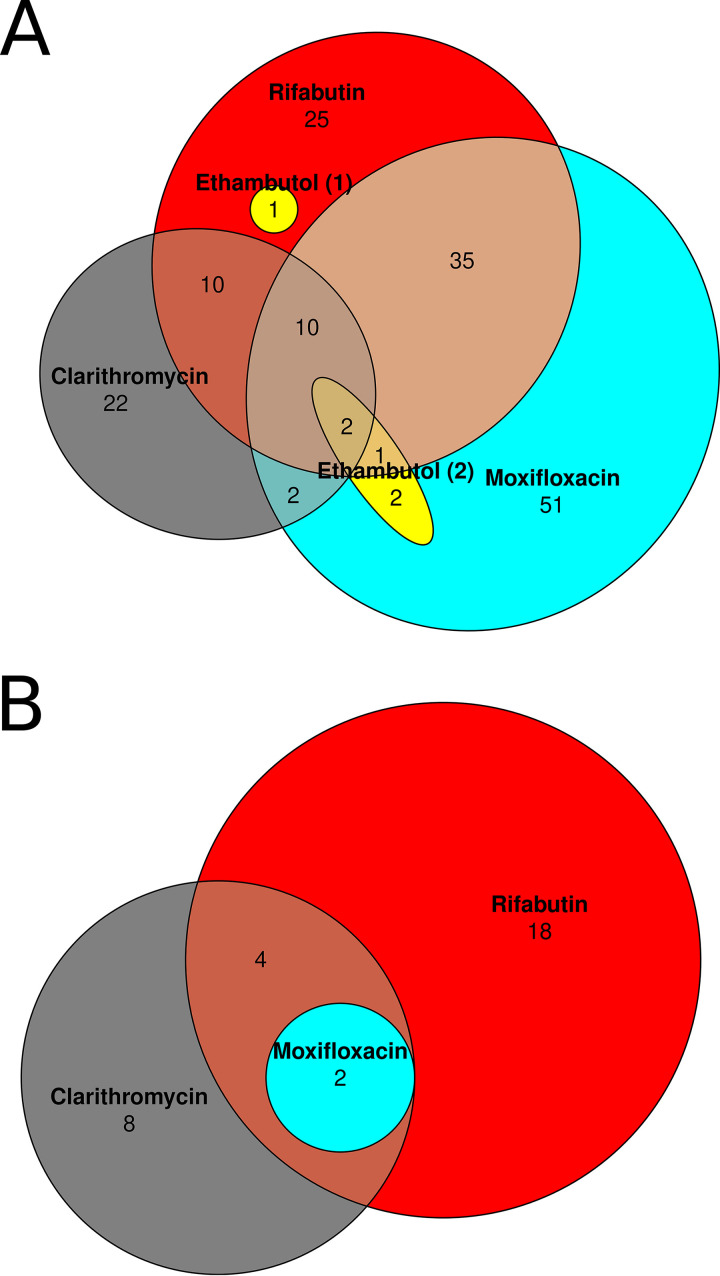
Venn diagram of identified hypersusceptible (A) and hypertolerant transposon mutants (B). Note that in A, the set of ethambutol-hypersusceptible mutants has been partitioned into two sets (both in yellow). Partitioning in this way greatly simplifies the diagram. Gene names in each category can be found in Table S1 and S2.

We also evaluated for overlaps between the different drug classes tested. Our results of this analysis are summarized in Fig. 2 and Table S1 (hypersusceptible mutants) and Table S2 (hypertolerant mutants) in the supplemental material. Mutants hypersusceptible to multiple antibiotics may reflect genes with a role in more general bacterial persistence mechanisms, while mutants hypertolerant to multiple antibiotics may suggest genes promoting antibiotic susceptibility. Notably, no mutant was found to be hypersusceptible to one antibiotic but hypertolerant to another.

Two mutants were hypersusceptible to all four antibiotics tested, namely, DFS55_00905 (annotated as an acyltransferase, homologous to M. tuberculosis Rv0111) and DFS55_12730 (hypothetical protein, homologous to Rv1836c). Ten mutants were hypersusceptible to CLR, MOX, and RFB, but not to EMB.

The following two mutants were identified as hypertolerant to CLR, MOX, and RFB (no mutant was hypertolerant to EMB): DFS55_10765 (annotated as a pyruvate kinase, homologous to Rv1617) and DFS55_20040 (DUF1707 domain-containing protein, homologous to Rv0966c). An additional 4 mutants were found to be hypertolerant to RFB and CLR only, but not to MOX. These mutants included DFS55_10660 (quinolinate synthase, homologus to Rv1594), DFS55_10665 (l-aspartate oxidase, homologous to Rv1595), DFS55_16845 (trigger factor, homologous to Rv2462c), and DFS55_21750 (hypothetical protein, homologous to Rv3489).

## DISCUSSION

In this work, we utilized a genome-wide transposon mutant pool to screen for M. avium mutants with altered susceptibility to various clinically relevant antibiotics. Compared with the other antibiotics, exposure to MOX yielded the highest number of hypersusceptible mutants, highlighting the many potential targets which might synergize with this antibiotic. Also, given the strong effect sizes observed with MOX relative to the other antibiotics tested, MOX synthetic lethality may represent the greatest opportunity for novel treatment-shortening strategies.

Transposon insertions in several known virulence genes were found to enhance the susceptibility of M. avium to multiple antibiotics. For example, mutations in *secA2* (DFS55_12665 or *rv1821*) conferred hypersusceptibility to both CLR and MOX. The Sec export pathway is conserved across bacteria and exports secreted proteins across the cytoplasmic membrane (37). Mycobacteria have two SecA proteins, namely, SecA1 and SecA2 (37). While SecA1 is essential and facilitates the transport of unfolded proteins though the SecYEG channel via its ATPase activity, the mechanism of export in the SecA2 pathway is less well understood (38). SecA2 is required for secretion of M. tuberculosis virulence proteins and arrest of phagosome maturation by preventing acidification, thereby facilitating M. tuberculosis growth within macrophages (39). In particular, the SecA2-secreted phosphatase SapM and the kinase PknG have been identified as effectors with direct roles in preventing phagosome maturation and promoting M. tuberculosis intracellular survival and replication (40). As CLR inhibits protein synthesis (41) and SecA2 disruption impairs the secretion of virulence-related proteins, these two alterations, which both dysregulate proteostasis, may have a synergistic or additive effect, leading to higher antibiotic susceptibility for this mutant. A similar but more indirect mechanism could be proposed for the sensitization of this mutant to MOX, which inhibits DNA gyrase and topoisomerase IV (41). Both of these enzymes are involved in the winding and unwinding of DNA and are necessary for DNA replication and RNA transcription (42, 43). MOX-induced reductions in mRNA transcripts may also dysregulate proteostasis in MAC, potentially explaining the similar phenotypes observed with CLR exposure.

RecA (DFS55_08530 or Rv2737c), which was found to be required for MAC tolerance to both MOX and RFB, plays a critical role in the mycobacterial DNA damage response, specifically in the repair of double-stranded breaks, as M. smegmatis cells lacking RecA are more sensitive to DNA damage (44). Specifically, after double-stranded breaks are resected by AdnAB, RecA is loaded onto the 3′ end of the DNA, helping to mediate a homology search and subsequent strand invasion (44). By inhibiting DNA topoisomerases, MOX promotes DNA damage and triggers a mutagenic SOS response, which can lead to the formation of persister cells (45). RecA activation promotes the self-cleavage of LexA leading to upregulation of the SOS regulon (46). In turn, removal of RecA leads to the suppression of the SOS regulon and decreased persister formation. Chemical inhibition of RecA with suramin in DNA gyrase-depleted cells has been shown to improve killing of M. tuberculosis by several anti-TB drugs, including rifampin and EMB (45). RFB, the rifamycin tested in our study, inhibits DNA-dependent RNA polymerase and suppresses RNA synthesis. Previous work showed that a *recA*-deficient M. tuberculosis mutant was unable to develop resistance to rifampin, possibly due to an inability to generate an SOS response (47). In short, it appears that inhibiting RecA, thereby suppressing the SOS response, could provide a means to decrease persister formation and improve killing of pathogenic mycobacteria, such as M. tuberculosis and MAC.

Interestingly, we identified two mutants as hypersusceptible to all four antibiotics tested in our study (Fig. 2). They included mutants with transposon insertions in DFS55_00905 (annotated as an acyltransferase, homologous to *rv0111*) and DFS55_12730 (hypothetical protein, homologous to *rv1836c*). Mutants in DFS55_00905 displayed particularly robust hypersusceptibility to MOX (effect size of −2.0 at 1 μg/ml and 48-h exposure) and EMB (effect size of −1.4 at 2.1 μg/ml, 48 h, which was the largest effect size we observed with this drug at 48 h). Mutants in DFS55_12730 were strongly hypersusceptible to CLR (effect size of-1.6 at 5.4 μg/ml and 48 h), MOX (−1.9 at 1.0 μg/ml and 48 h), and RFB (−1.5 at 0.63 μg/ml and 48 h). Future work should investigate the function of these gene products and their relationship to the pansusceptibility phenotype observed.

An additional 10 mutants were found to be hypersusceptible to CLR, MOX, and RFB, but not to EMB. They included mutants in *sigE* (DFS55_18590, *rv1221*) and an alpha-beta hydrolase gene (DFS55_15065, homologous to *rv2224c*, also known as *caeA* or *hip1*). Deficiency of sigma factor E (SigE) has been shown to confer increased susceptibility of M. tuberculosis to multiple drugs, including EMB and rifampin, but not to ciprofloxacin (48). These results differ somewhat from the results of our study. Upon closer examination of our results for EMB, we find that this mutant was barely outside our conservative thresholds for defining hypersusceptible mutants. While adjusted *P* values for EMB at both time points were below the cutoff (0.045 and 0.0001; cutoff, 0.05) (see Table S6), the corresponding log-fold changes were barely above our chosen thresholds (−0.47 and −1; cutoff, −0.5) (Table S6). Therefore, it is possible that our stringent cutoffs misclassified this mutant as displaying a similar EMB susceptibility as the wild type. On the other hand, the discrepancy regarding hypersusceptibility of *sigE*-deficient mycobacteria to fluoroquinolones may be due to differences between the two species (M. tuberculosis versus MAC, which have substantially different growth rates) and/or the experimental designs of the two studies (resazurin microtiter assay versus Tn-seq screen). Additional studies are required to further evaluate the impact of *sigE* deficiency on MAC susceptibility to EMB and fluoroquinolones. Consistent with our data in MAC, M. tuberculosis mutants in *caeA*/*hip1*/*rv2224c* have been shown to be hypersusceptible to rifamycins (rifampin) (36, 49) and macrolides (erythromycin) (49). Our data suggest that *caeA* (DFS55_15065) deficiency also confers enhanced susceptibility to fluoroquinolones (Table S1 and S4), which might be useful for designing novel therapies for M. tuberculosis.

Xu et al. screened a M. tuberculosis transposon mutant pool exposed to EMB, the only antibiotic shared with our study, and identified 45 hypersusceptible transposon mutants (*q* value of <0.05) (36). While the species of organism used and experimental design of our study differ from those of Xu et al., we were intrigued to discover that 3 of the 6 genes meeting our stringent cutoffs (DFS55_00120/*rv0019c*, DFS55_03885/*rv0642c*, and DFS55_12730/*rv1836c*) were shared by both lists of hypersusceptible mutants. This large fraction of genetic overlap suggests there may be some common genetic elements that lead to EMB hypersusceptibility in mycobacteria more generally.

We also identified two mutants with hypertolerance to three of the four antibiotics tested (CLR, MOX, and RFB), namely, DFS55_10765 (annotated as a pyruvate kinase, *rv1617*) and DFS55_20040 (DUF1707 domain-containing protein, *rv0966c*). Interestingly, Rv1617 deficiency is associated with a large growth defect in M. tuberculosis (34, 35), but the same phenotype is not observed in DFS55_10765-deficient M. avium (35). This result suggests that the metabolic impact of pyruvate kinase deficiency is remarkably different between MAC and M. tuberculosis. Pyruvate kinase catalyzes the transfer of a phosphate group from phosphoenolpyruvate to ADP (yielding pyruvate and ATP). Central metabolism may be disrupted in bacteria lacking this enzyme, possibly triggering the stringent response, which has been previously shown to protect bacteria from antibiotic-mediated killing (50, 51). Deletion of pyruvate kinase in M. tuberculosis causes allosteric inhibition of the trichloroacetic acid (TCA) cycle through accumulation of phosphoenolpyruvate (52). Disruption of the TCA cycle, especially alternate catabolism through the glyoxylate shunt, has been linked to antibiotic tolerance in multiple bacterial species, including Pseudomonas aeruginosa (53), Staphylococcus aureus (54, 55), and Staphylococcus epidermis (56), suggesting that this pathway may be a common mechanism for promoting antibiotic tolerance. In M. tuberculosis, downregulation of the malate synthase GlcB, one of the enzymes in the glyoxylate shunt, caused increased susceptibility to both rifampin and to nitrosative and oxidative stresses *in vitro* (57). Deficiency of isocitrate lyase, another glyoxylate shunt enzyme, also led to increased susceptibility of M. tuberculosis to antibiotics *in vitro* (58) and decreased survival in activated macrophages and mice (27). Thus, mycobacterial metabolism, and the TCA cycle in particular, clearly plays an important role in the development of antibiotic tolerance, although more work is necessary to fully elucidate its contributions. DFS55_20040 appears to lack an annotated function in the literature. Additional work is needed to understand the function of these two genes and determine their relationship to antibiotic tolerance in mycobacteria.

Our approach has several limitations. First, mutants in essential genes (or those without TA insertion sites) could not be screened, as they could not be recovered with our bacterial regrowth techniques. Therefore, we were unable to assess the potential role in antibiotic tolerance of genes essential for growth of M. avium in nutrient-rich medium. Second, gene disruptions leading to changes in secreted factors (e.g., extracellular proteins) may have been missed by our screen, as these factors may be complemented by factors produced by nondefective mutants present in the same culture. Third, we chose a conservative statistical approach (JT-test) and conservative thresholds for *P* values and log-fold changes (LFCs), which must be met at two different time points. It is likely that mutants with low numbers of insertion sites or somewhat weaker effect sizes were missed. Lastly, we have performed these screens in only a single strain of M. avium (MAC109), and it remains to be determined to what extent our data apply to other M. avium strains.

As we noted above, some clumping was observed in cultures at the 12-h time point, reducing the usefulness of the CFU measure but unlikely to impact the loss of hypersusceptible mutants. Tween 80 is a synthetic detergent known to greatly reduce clumping in some other mycobacterial species, such as M. tuberculosis. However, inclusion of this detergent is known to strongly impact antibiotic susceptibility in mycobacteria, including M. avium (59). Given that our preliminary culture work displayed limited clumping using medium without Tween 80, we decided to exclude it from our experiments (data not shown). It is possible that the addition of this detergent could reduce the clumping we observed at 12 h, although we did not test this possibility.

Previous studies have examined mycobacterial antibiotic hypersusceptibility in the context of very low antibiotic concentrations (36). In such an experimental setup, the entire bacterial population continues to grow during antibiotic exposure, and libraries are generated directly from bacterial cultures. In contrast, our approach here uses an additional regrowth step on solid agar after antibiotic exposure. This regrowth step produces sufficient material for library generation independent of whether the aggregate bacterial population is growing, stable, or dying. Therefore, our approach is more generally applicable to clinical scenarios in which higher doses of antibiotics may be used, inhibiting aggregate mycobacterial growth.

Mutations causing defects while the aggregate population declines or is static are interpreted in our screen as amplifying the killing effect of the antibiotic (given that the wild-type organisms can be assumed to be nongrowing). However, an observation of hypersusceptibility in the context of aggregate growth is more difficult to precisely resolve. Thus, it could be that the mutant is killed in the presence of the antibiotic, whereas the wild type is able to grow, or it is possible that the mutant is more inhibited by the antibiotic than the wild type but continues to grow, albeit at a lower rate. In particular, the overall population declined in the presence of 5.4 μg/ml CLR, suggesting that any defective mutants are killed more rapidly than the wild type. However, at 0.54 μg/ml CLR, the overall population increased, suggesting either that defective mutants could be killed more rapidly or merely that their growth is inhibited to a greater extent than that of wild type (see Fig. S2). Follow-up studies are needed in order to resolve the behavior of hypersusceptible mutants at this dose.

Our study represents a first step toward the development of novel, treatment-shortening strategies for MAC infections through identification of genes mediating antibiotic susceptibility. Biochemical characterization of the corresponding gene products might yield novel insights into the mechanisms of MAC antibiotic tolerance and lay the groundwork for the development of novel antibiotics, which might synergize with currently available drugs to kill tolerant organisms and shorten curative treatment for MAC infections more effectively. Future work is needed to validate the susceptibility phenotypes of individual gene-deficient mutants and their respective complemented strains in axenic cultures. Proof-of-concept studies could then be performed to demonstrate the treatment-shortening potential of candidate targets in a relevant animal model of pulmonary MAC disease (60, 61).

## MATERIALS AND METHODS

### Strains.

All experiments were performed using Mycobacterium avium subsp. *hominissuis* strain 109 (35).

### Media and buffers.

To make 7H11 agar, 10.25 g of 7H11 without malachite green powder (catalog [cat] no. 511A; HiMedia) was added to 450 ml deionized water. A volume of 5 ml 50% glycerol was added and then the mixture was autoclaved. The agar was cooled to 55°C before addition of 50 ml oleic acid-albumin-dextrose-catalase (OADC) enrichment (Becton, Dickinson). To make 7H9-30% OADC, 2.35 g of 7H9 powder was added to 350 ml deionized water. After sterilization (by autoclaving at 121°C or passing through a 0.22-μm filter), 150 ml of OADC enrichment was added. Unless otherwise specified, no Tween 80 or glycerol was included in the media. To make phosphate-buffered saline with Tween 80 (PBS-T), 1.25 ml of filter-sterilized 20% Tween 80 was added to 500 ml of sterile PBS.

### Overview of transposon screen setup.

**FIG 3:**
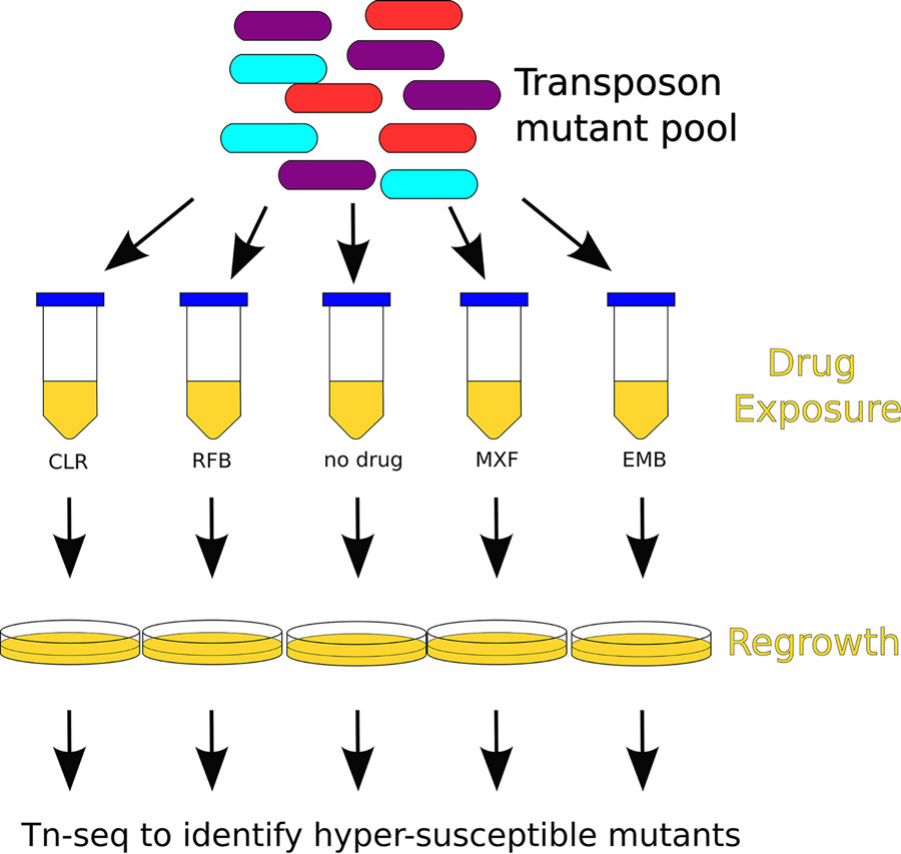
Schematic of transposon mutant screen to identify hypersusceptible mutants following exposure to multiple doses of various antibiotics in liquid culture. After antibiotic exposure, cultures were regrown on solid agar to enrich for surviving bacteria. After regrowth, DNA was extracted and prepared for Tn-seq analysis. Hypersusceptible mutants were identified using a nonparametric statistical approach.

The setup for our genome-wide differential susceptibility screen is shown in Fig. 3. We inoculated two 1-ml frozen aliquots of a previously generated diverse *Himar1* transposon mutant pool (consisting of approximately 1.2 × 10^6^ unique mutants each) (35) into two 1.3-liter roller bottles each containing 250 ml of 7H9-30% OADC. The bottles was shaken overnight at 37°C to allow for some growth and reduce bacterial clumping (190 rpm, 0.75-in [1.905 cm] orbit). The optical density at 600 nm (OD_600_) was tracked until reaching 0.2 and 0.22. The bottles were then pooled, diluted to OD_600_ of 0.1 with cold 7H9-30% OADC, and aliquoted into 89 50-ml conical tubes (10 ml/tube). Tubes were incubated for 5 hours while shaking at 37^°^C to allow the bacteria to return to log-phase growth. Five tubes were randomly selected for processing at 0 hours. These samples were then processed for colony forming unit (CFU) enumeration and regrowth. The same tube was used for both operations. Following regrowth, bacterial samples were scraped, the DNA was extracted, and libraries were prepared for Tn-seq. Bacterial regrowth is required before Tn-seq library preparation in order to remove the contribution of DNA to the sequencing library from dead transposon mutants. After collection of enumeration and regrowth of samples at 0 hours, antibiotics (or dimethyl sulfoxide [DMSO] vehicle as a control) were then added to the remaining 84 tubes. Samples were collected at 12 and 48 hours after antibiotic exposure in triplicate and processed for CFU enumeration, bacterial regrowth, and Tn-seq library preparation in the same manner as the 0-hour samples.

### Sample processing for CFU enumeration and regrowth.

The bacterial density (CFU/ml) was estimated by removing 400 μl of bacterial culture, centrifuging (2,000 × *g* for 5 minutes), and washing twice with PBS-T to remove the antibiotic. Washed samples were diluted 10-fold, and 50 μl of each dilution was plated onto 7H11 agar without malachite green. Malachite green, which is typically present in standard 7H10 and 7H11 agar formulations, was excluded to avoid potential issues with postantibiotic recovery in mycobacteria (62). T-shaped spreaders were used to spread liquid evenly across agar plates. CFUs were counted after 7 to 8 days.

For bacterial regrowth, the remainder of each tube (after removing samples for CFU enumeration) was centrifuged twice and washed (2,000 × *g* for 10 min) with 10 ml of PBS-T to remove the antibiotic. The samples were centrifuged once more, and the bacterial pellet was resuspended in 250 μl PBS-T. Fifty microliters of the washed transposon pool was plated onto each of four 7H11 agar plates and spread with 10 to 15 3-mm sterile glass beads to ensure even distribution of liquid across the plate. Samples were regrown for 7 to 8 days. Bacterial lawns from the four agar plates were scraped and pooled into 2-ml tubes. DNA was extracted from regrown samples, as described previously (short-read sequencing protocol) (63). DNA was processed for Tn-Seq, as described previously (35). Libraries were sequenced (2 × 75 bp) on an Illumina HiSeq 2500 instrument by the Johns Hopkins Genetic Resources Core Facility (GRCF) High Throughput Sequencing Center. A total of 59 samples (5 input-pool samples and 18 groups of output-pool triplicates) were sequenced, yielding between 2,333,295 and 7,193,522 reads per sample for a total of 269,324,560 paired-end reads.

### Antibiotic selection.

Doses were selected to reflect antibiotic concentrations at the most common site of infection in noncompromised patients (the lungs) following standard antibiotic dosing. Based on a search of the pharmacokinetic literature, maximum achievable doses in lung tissues were taken to be 54 μg/ml for CLR (64), 0.63 μg/ml for RFB (65), 10.0 μg/ml for MOX (66), and 21.0 μg/ml for EMB (67) (based on nonhuman primate data). For each drug, a 10-fold dilution series of the estimated maximum achievable dose was performed to explore the impact of dose.

Before setting up the cultures used for this study, a preliminary calibration experiment (single replicate for each antibiotic, not processed for sequencing) was performed to estimate the bacterial viability at different antibiotic concentrations (data not shown) and to select the number of concentrations to include in the study (4 total concentrations for CLR, 3 concentrations for the other antibiotics in addition to drug-free controls).

### Sample selection for sequencing from susceptibility screen.

A subset of samples from the differential susceptibility screen was chosen for Tn-seq library prep and sequencing. Samples were sequenced at two manually chosen concentrations for each drug at both available time points (12 h and 48 h). To help identify which should be processed further, objective criteria were established *a priori*, with the goal of identifying mutants with higher susceptibility to antibiotics but also reducing the likelihood that mutants were removed by chance due to low bacterial viability during antibiotic exposure. We considered the following 3 criteria for selecting samples:

- Bacterial numbers must exceed 10^6^ CFU/ml at all times during exposure. This ensures that the probability of losing a nondefective mutant is minimized, given the 60,129 possible thymine-adenine (TA) sites for the *Himar1* transposon to insert across the MAC109 genome. Only CFU data were used, and we did not attempt to directly estimate the total number of bacterial cells, which can differ significantly from CFU.
- There should be decreased bacterial viability after antibiotic exposure relative to drug-free controls (as measured by CFU). This ensures that the concentration of antibiotic is high enough to have bactericidal activity. Otherwise, the antibiotic concentration might be too low to select for or against mutants with growth phenotypes.
- Drug concentrations near or below achievable serum concentrations of the drug after standard dosing are preferred. We assumed approximate serum values of 2.31 μg/ml for CLR, 4.42 μg/ml for MOX, 0.52 μg/ml RFB, and 2.27 μg/mL for EMB (68). In our view, this criterion makes the results more clinically relevant.

### Identification of hypersusceptible mutants from sequencing data.

A schematic of the pipeline to process the data is provided in Fig. S1 in the supplemental material. Raw reads were mapped using the TRANSIT preprocessor (tpp) (69). Counts from tpp were then processed with a custom python script to produce a *.csv file to be read by pandas (version 0.24.1) used for downstream analyses.

### Effect size/log-fold change calculation.

For calculation of the normalized read counts for each mutant, a pseudocount (70) of 4 was added to the raw count from all samples before dividing read counts by total read count:

$${\tilde{x}}_{{}_{t,\mathrm{ir}}}=\frac{X_{{}_{t,\mathrm{ir}}}+\alpha }{\sum_{t}^{T}\left(X_{{}_{t,\mathrm{ir}}}+\alpha \right)}$$

where *˜x_t_*_,_*_ir_* is the normalized read count; *X_t_*_,_*_ir_* is the raw read count for transposon insertion site *t*, for antibiotic treatment group *i*, and for replicate *r*. *α* is the pseudocount (*α* = 4); and *T* is the number of transposon insertion sites. This pseudocount was added to stabilize the normalized count and was determined by manual examination of read counts in genes known to be essential. The pseudocount was set to be substantially larger than occasional background reads. The normalized read counts were then averaged over samples:

$$\mu_{t,i}=\frac{1}{n_{i}}\sum_{r}^{n_{i}}{\tilde{x}}_{{}_{t,\mathrm{ir}}}$$

where *μ_t_*_,_*_i_* is the average representation of each mutant across samples and *n_i_* is the number of replicates for treatment group *i*. An aggregate log-fold change (LFC) was used as a measure of effect size for differentially susceptible mutants. The aggregate LFC between treatment groups *i* and *j* was calculated as the median of the log-fold change at individual transposon insertion sites within a gene:

$$LFC_{g,\left(i/j\right)}={\text{med}}_{t\in G_{g}}\left[\log_{2}\left(\frac{\mu_{t,i}}{\mu_{t,j}}\right)\right]$$

where *G_g_* is the set of transposon insertion sites annotated to belong to gene *g* and *LFC_g_*_,_*_(i/j)_* is the log-fold change between treatment groups *i* and *j* for gene *g*. Although this formula is generally useful for comparing any pair of groups, for the work presented here, *i* always represents a drug-containing treatment group and *j* always represents the matching drug-free group at the same time point.

### *P* value calculation.

For the calculation of *P* values, read counts for each sample were first normalized by dividing by the total read count in each sample (pseudocounts are unnecessary for the nonparametric test described below and thus were not used).

$$x_{{}_{t,\mathrm{ir}}}=\frac{X_{{}_{t,\mathrm{ir}}}}{\sum_{t}^{T}X_{{}_{t,\mathrm{ir}}}}$$

The Jonckheere-Terpstra (JT) test was then applied to the normalized read counts at each time point (71, 72). Briefly, the JT test is a nonparametric test of trend, which is more powerful than the more commonly used Kruskal-Wallis test when the alternative hypothesis assumes a monotonic trend of the treatment groups. In this case, we have three treatment groups for each drug at each time point, namely, no drug, low dose, and high dose. We postulated that if a mutant is hypersusceptible at a low dose of antibiotic, it will be even more hypersusceptible at a higher dose of that antibiotic. We define the alternative hypothesis as:

$$H_{A}:\theta_{1}\leq \theta_{2}\leq \ldots \leq \theta_{K}$$

against a null hypothesis:

$$H_{0}:\theta_{1}=\theta_{2}=\ldots =\theta_{K}$$

where *θ*_1_*…θ_K_* are measures of a centrality parameter and *K* is the number of treatment groups for the drug at a particular time point (in this case *K* = 3 for each drug). The JT test statistic (*B_t_*) at site *t* is defined as:

$$B_{t}=\sum_{i=1}^{K-1}\sum_{j=i+1}^{K}\sum_{r=1}^{n_{i}}\sum_{s=1}^{n_{j}}1\left[x_{t,\mathrm{ir}}<x_{t,js}\right]$$

(where 1[] is the indicator function). *P* values at individual sites are computed via permutation test. Naively, this would result in nonuniformly distributed *P* values due to the discrete nature of the distribution. To ensure a truly uniform distribution, we performed a small correction on the permutation test *P* values by sampling from a uniform distribution bounded between adjacent values of the discrete permutation distribution. This process gives precisely uniform *P* values under the null hypothesis.

Pooling the *P* values within each gene (one *P* value calculated for each transposon insertion site) is then accomplished with the two-sided Stouffer’s method. Finally, adjusted *P* values are then computed using the Benjamini-Hochberg procedure.

### Thresholds for defining differentially susceptible mutants.

Gene mutants are considered differentially susceptible to a drug if the absolute value of the LFC at the high dose of each drug (relative to no drug control) is greater than 0.5 and the adjusted *P* value is less than 0.05. These conditions must be met at both the 12-hours and 48-hour time points in order for the mutant to be defined as differentially susceptible to the drug. Mutants with negative LFC are predicted to be hypersusceptible to the drug, while a positive LFC indicates the mutant is hypertolerant.

### Data availability.

The raw sequencing data (*.fastq) from this project can be found in the NCBI Sequence Read Archive (SRA) under BioProject number PRJNA559896. A Jupyter notebook and associated scripts to reproduce the data analysis (including figures) from the raw data are provided online at github (https://doi.org/10.5281/zenodo.4542412).
