# Supplementary material for: Genetic Determinants of Intrinsic Antibiotic Tolerance in Mycobacterium avium
Source: Microbiol Spectr. 2021 Sep 15;9(2):e00246-21. doi: 10.1128/Spectrum.00246-21 (PMC8557931; doi:10.1128/Spectrum.00246-21)
Supplement: SUPPLEMENTAL FILE 7 — Supplemental material. Download SPECTRUM00246-21_Supp_7_seq13.pdf, PDF file, 0.5 MB [file spectrum00246-21_supp_7_seq13.pdf]

Supplementary Figures and Captions

Supplementary Table Captions

Table S1**:** Summary prediction table for antibiotic hypersusceptible MAC109 mutants.

Table S2: Summary prediction table for antibiotic hypertolerant MAC109 mutants.

Tables S3**:** Clarithromycin-hypersusceptible and hypertolerant MAC109 transposon mutants by dose and time point.

Tables S4**:** Moxifloxacin-hypersusceptible and hypertolerant MAC109 transposon mutants by dose and time point.

Tables S5**:** Rifabutin-hypersusceptible and hypertolerant MAC109 transposon mutants by dose and time point.

Tables S6**:** Ethambutol-hypersusceptible and hypertolerant MAC109 transposon mutants by dose and time point.

Supplementary Figures

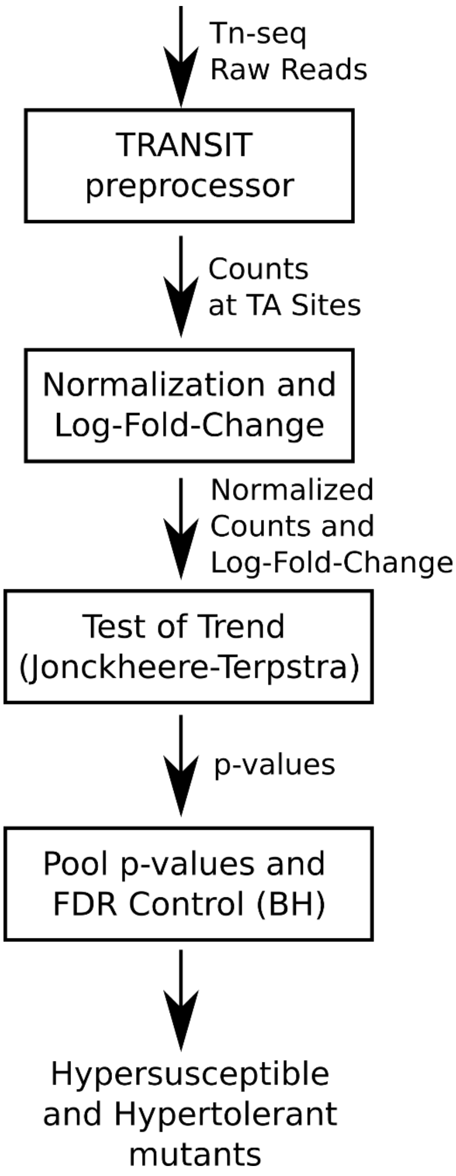

Figure S1**:** Schematic of the computational pipeline. The raw reads from Tn-seq were input into tpp, outputting a count for each TA position in the MAC109 genome. Counts were then normalized and log-fold changes calculated. A non-parametric test of trend (Jonckheere-Terpstra Test) was used to calculate p-values for each TA site. P-values for TA sites in the same gene were pooled using Stouffer's method. Statistically significant mutants were selected based on the log-fold changes and Benjamini-Hochberg FDR-adjusted p-values. See Methods for additional details of these steps.

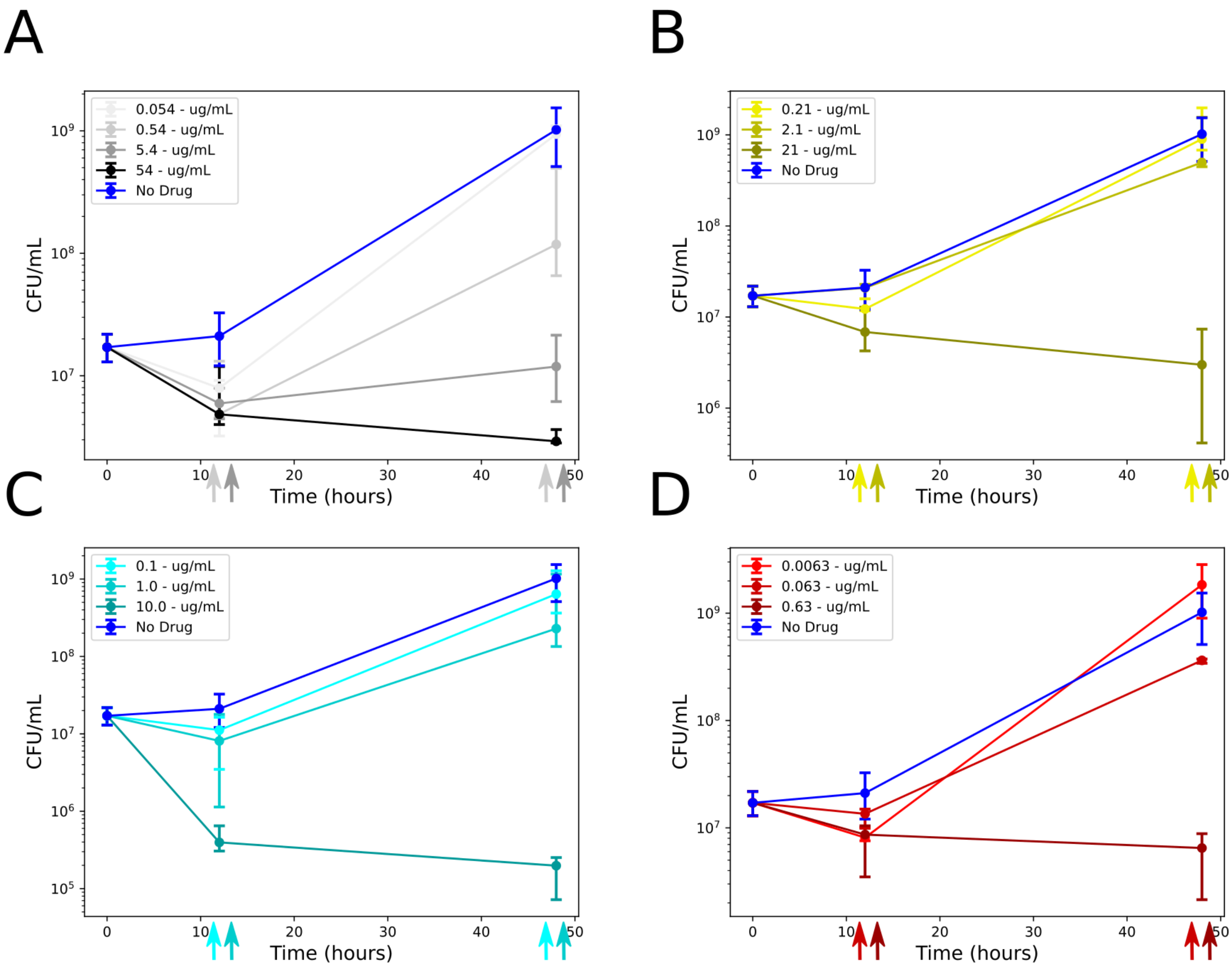

Figure S2: Bacterial viability of cultures by drug, dose, and time point (3 replicates each). Error bars show the minimum, maximum, and median. The arrows at the bottom indicate time point and doses used for Tn-seq. Drug-free controls (blue) are replotted in each subpanel for reference. All doses and time points were collected for drug-free controls. (A) Clarithromycin. (B) Ethambutol. (C) Moxifloxacin. (D) Rifabutin.

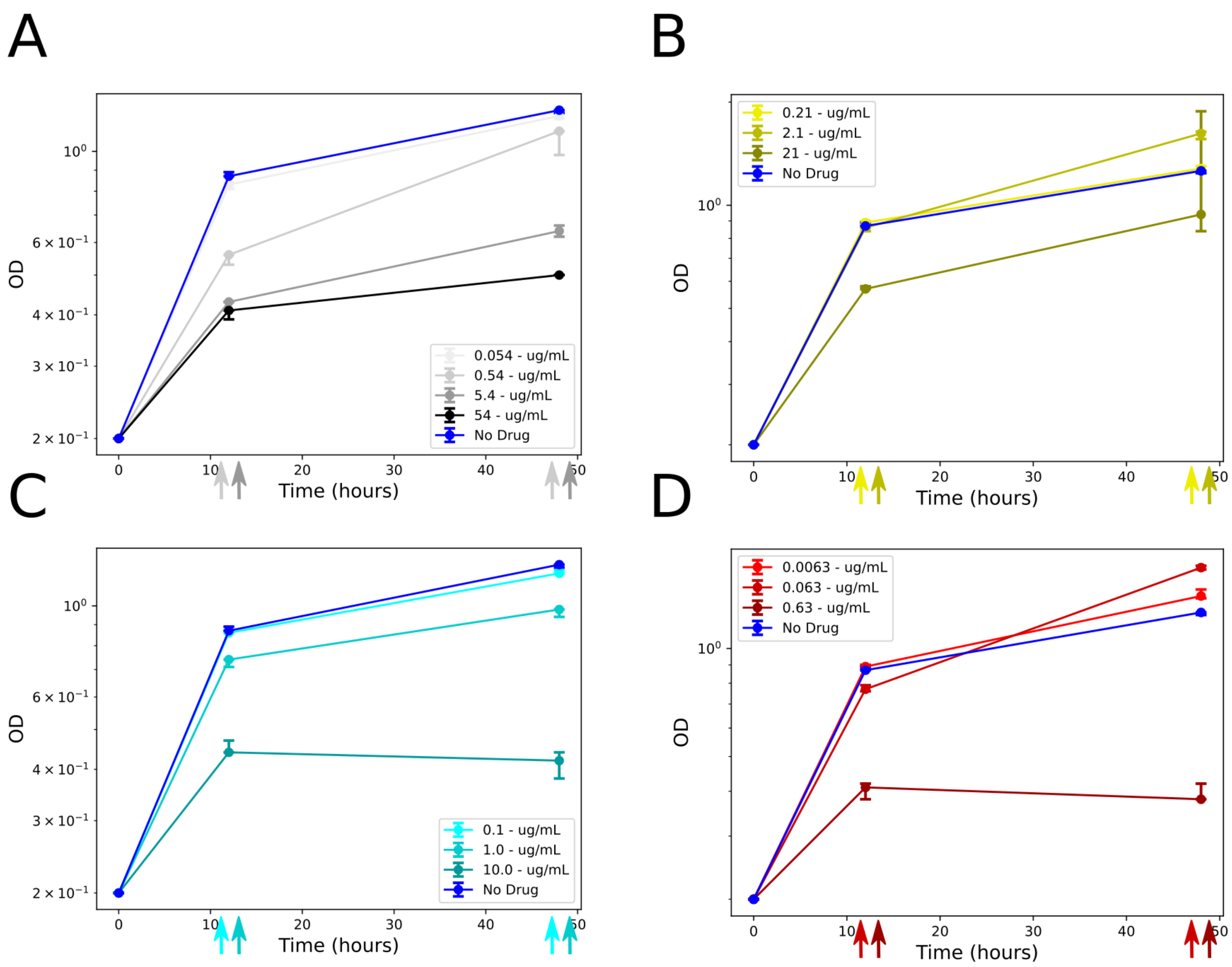

***Figure S3*:** OD_{600} values by drug, dose, and time point. The arrows at the bottom indicate time point and doses used for Tn-seq. Drug-free controls (blue) are replotted in each subpanel for reference. All doses and time points were used for drug-free controls. (A) Clarithromycin. (B) Ethambutol. (C) Moxifloxacin. (D) Rifabutin.
